# Head and neck region consolidation radiotherapy and prophylactic cranial irradiation with hippocampal avoidance delivered with helical tomotherapy after induction chemotherapy for non-sinonasal neuroendocrine carcinoma of the upper airways

**DOI:** 10.1186/1748-717X-7-21

**Published:** 2012-02-15

**Authors:** Pierfrancesco Franco, Gianmauro Numico, Fernanda Migliaccio, Paola Catuzzo, Domenico Cante, Paola Ceroni, Piera Sciacero, Pierpaolo Carassai, Paolo Canzi, Maria Rosa La Porta, Giuseppe Girelli, Valeria Casanova Borca, Massimo Pasquino, Santi Tofani, Franca Ozzello, Umberto Ricardi

**Affiliations:** 1 Radiation Oncology Department, Tomotherapy Unit, Ospedale Regionale 'U.Parini', AUSL Valle d'Aosta, Viale Ginevra n° 3, 11100 Aosta, Italy; 2 Medical Oncology Department, Ospedale Regionale 'U.Parini', AUSL Valle d'Aosta, Viale Ginevra n° 3, 11100 Aosta, Italy; 3 Medical Physics Department, Ospedale Regionale 'U.Parini', AUSL Valle d'Aosta, Viale Ginevra n° 3, 11100 Aosta, Italy; 4 Pathology Department, Ospedale Regionale 'U.Parini', AUSL Valle d'Aosta, Viale Ginevra n° 3, 11100 Aosta, Italy; 5 ENT Department, Ospedale Regionale 'U.Parini', AUSL Valle d'Aosta, Viale Ginevra n° 3, 11100 Aosta, Italy; 6 Radiotherapy Department, ASL TO4, Ospedale Civile di Ivrea, Ivrea, Italy; 7 Medical Physics Department, ASL TO4, Ospedale Civile di Ivrea, Ivrea, Italy; 8 Department of Medical and Surgical Sciences, Radiation Oncology Unit, University of Torino, Ospedale San Giovanni Battista, Turin, Italy

**Keywords:** Radiotherapy, Tomotherapy, Non-sinonasal neuroendocrine carcinoma, Head and neck, Hippocampus avoidance, Prophylactic cranial irradiation

## Abstract

**Background:**

Non-sinonasal neuroendocrine carcinomas (NSNECs) of the head and neck are considered an unfrequent clinico-pathological entity. Combined modality treatment represents an established therapeutic option for undifferentiated forms where distant metastasis is a common pattern of failure.

**Methods:**

We report on a case of NSNEC treated with sequential chemo-radiation consisting of 6 cycles of cisplatin and etoposide followed by loco-regional radiation to the head and neck and simultaneous prophylactic cranial irradiation to prevent from intracranial spread, delivered with helical tomotherapy with the 'hippocampal avoidance' technique in order to reduce neuro-cognitive late effects.

**Results:**

One year after the end of the whole combined modality approach, the patient achieved complete remission, with no treatment-related sub-acute and late effects.

**Conclusions:**

The present report highlights the importance of multidisciplinary management for NSNECs of the head and neck, as the possibility to achieve substantial cure rates with mild side effects with modern radiotherapy techniques.

## Introduction

Tumours of neuroendocrine differentiation arising within the head and neck region are considered an extremely rare clinico-pathological entity [1]. They have been described in several anatomical sites such as upper airways (trachea, larynx, nose, paranasal sinuses), ear, tongue and salivary glands [2,3]. Some Authors have proposed a frame distinction between sinonasal neuroendocrine carcinomas (SNNECs) and non-sinonasal neuroendocrine carcinomas (NSNECs) in terms of pathological classification and therapeutic options [1,4]. SNNEC are divided into 4 main histological categories, namely esthesioneuroblastoma, undifferentiated carcinoma, neuroendocrine carcinoma and small cell undifferentiated carcinoma and might deserve a multimodality treatment approach regardless of their differentiation [1,4]. Conversely, NSNECs are represented by undifferentiated (small cell or large cell subtypes), moderately (atypical carcinoid) and well-differentiated (typical carcinoid) carcinomas [1,5]. They mainly arise within the larynx (where they represent 0.6-1% of all epithelial cancers), particularly in the epiglottis and supraglottic region (aryepiglottic folds and arytenoids) [6]. They predominantly affect males, smokers and present with locally advanced node positive disease [1,7]. Conservative surgery (where possible) might be considered adequate for well-differentiated subtypes, while combined modality treatment (chemotherapy and radiotherapy) is considered a mainstay option for undifferentiated forms where distant metastasis represent the major pattern of failure [1]. We herein report on a case of NSNEC of unknown primary site treated with a sequential chemo-radiation approach consisting of 6 cycles of cisplatin (DDP) and etoposide (VP-16) followed by loco-regional radiation to the head and neck region and simultaneous prophylactic cranial irradiation (PCI) to prevent from intracranial spread, delivered with helical tomotherapy (HT) with the 'hippocampal avoidance' (HA) technique in order to reduce radiation-induced neuro-cognitive late effects [8].

## Case report

**Figure 1:**
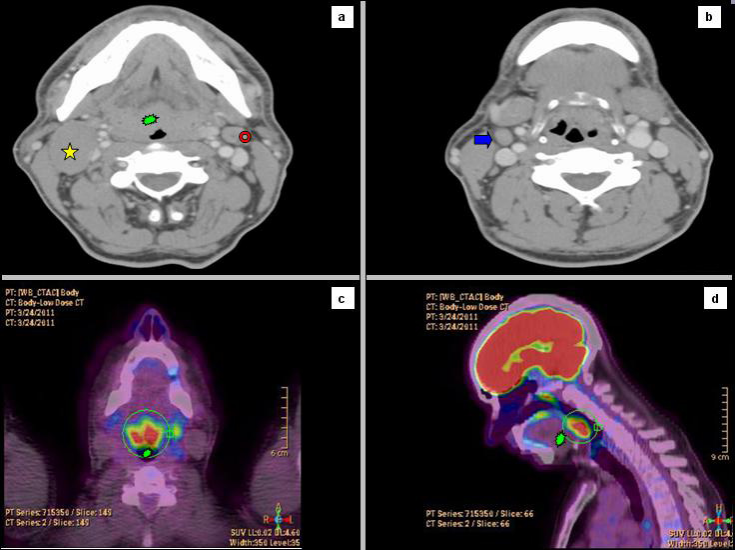
**Enlarged level Ib (star) and IIA (arrow) right nodes (Figure 1a-b) and level IIA left node (circle; Figure 1a) with thickening of the base of the tongue (blast; Figure 1a) at diagnostic CT scan; base of the tongue hyperaccumulation at 18-FDG- PET scan (Figure 1c-d)**.

**Figure 2:**
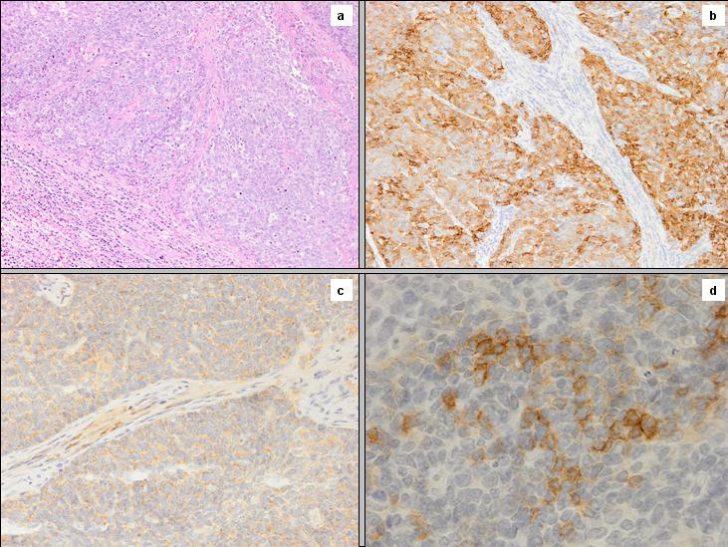
**Oat cells pattern at hematoxylin-eosin staining (Figure 2a); immunohistochemistry positive staining for AE1 and AE3 Cytokeratin (Figure 2b), Chromogranin A (Figure 2c) and CD 56 (Figure 2d)**.

**Figure 3:**
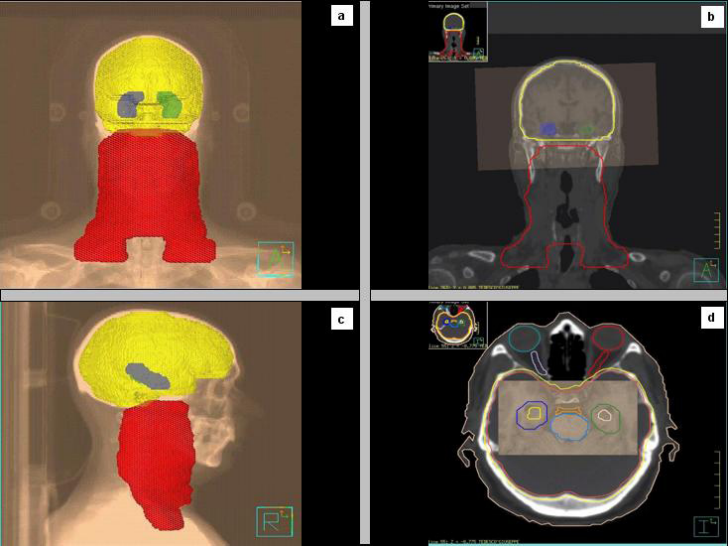
**Target volumes including the head and neck region and the whole brain with concomitant sparing of the bilateral hippocampal regions (Figure 3a-c); fusion MRI employed for appropriate delineation of the hippocampus (Figure 3b-d)**.

**Figure 4:**
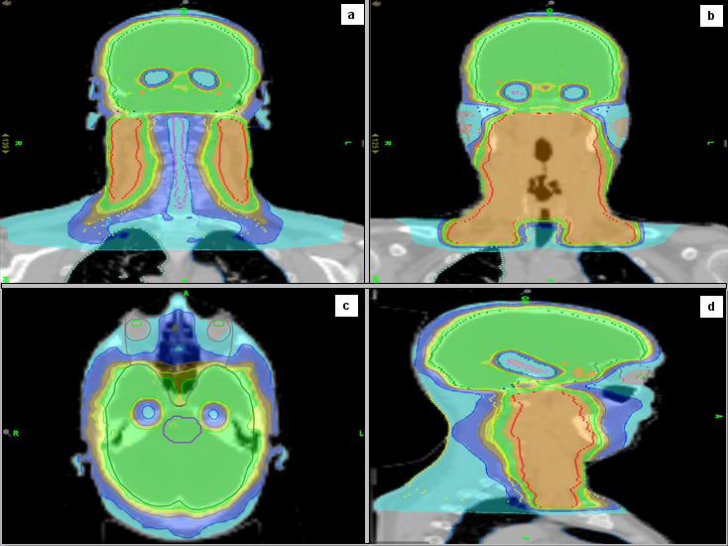
**Planning results in terms of isodoses distribution with organs at risk sparing, namely hippocampus (Figure 4a-d), spinal cord (Figure 4a), parotid glands (Figure 4b), ocular bulbs and lens (Figure 4c)**.

**Figure 5:**
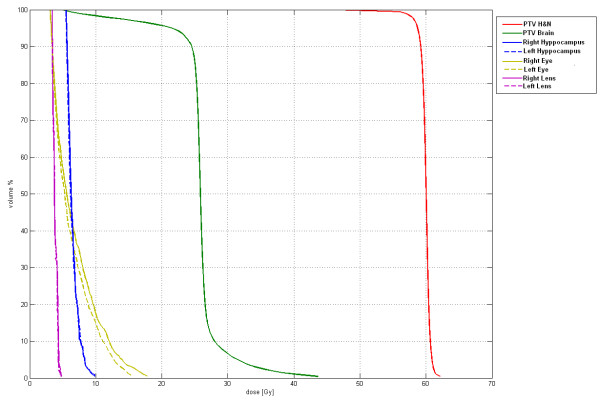
**Dose-volume histogram for target volumes and main intracranial organs at risk**.

A 53 years old caucasian man was referred to our Institution Hospital due to the sudden appearance of a right latero-cervical enlarged lymphnode with no symptoms complained. He had a previous medical history of childhood tonsillectomy, appendectomy, acute bacterial epididymitis and asymptomatic hepatitis A infection. He was a non-smoker and had a low-moderate alcohol intake at meals. Physical examination of the neck region showed a 3-cm hard and fixed adenopathy close to the posterior belly of the right digastric muscle. He underwent, at first, a pharyngo-laryngoscopy procedure that revealed a macroscopic tongue tonsil hypertrophy. A total body CT-scan demonstrated two enlarged lymphnodes (35 and 12 mm in diameter) in the right upper neck between the sub-mandibular group (level Ib) and the upper anterior jugular group (level IIA) according to Robbins classification with an adjunctive level IIA left node (15 mm in diameter) [9]; thickening of the base of the tongue could also be observed (Figure 1). He underwent an excisional biopsy of the right neck and a punch biopsy of the base of the tongue; histological findings of the lymphnode specimen documented undifferentiated small cell carcinoma (typical oat cells pattern; positive staining for AE1 and AE3 Cytokeratin, Chromogranin A and CD 56) with a Ki67 labelling index of 80%; base of the tongue was negative for tumour cells (Figure 2). For staging purposes a ^18 ^F-deoxyglucose- CT-positron emission tomography (CT-PET scan) was performed showing focal uptake within the oropharynx and left neck (Figure 1). Using flexible fiber-optic endoscopy he underwent directed bilateral biopses of the most likely primary tumour sites (nasopharynx, tongue base, tonsils, piriform sinus) with negative findings. Adjunctively a lingual tonsillectomy was performed with the evidence of hyperplastic lingual tonsillitis. At the end of diagnostic work-up: small cell undifferentiated NSNEC of unknown primary site (AJCC-UICC stage cTxN2bM0) was pointed out. Multimodality therapeutic approach was chosen consisting of induction CT followed by consolidation radiation; 6 cycles of the PE regimen were planned (Cisplatin 75 mg/m^2 ^day1 and Etoposide 100 mg/m^2 ^days 1,2,3 every 21 days). Intermediate CT and PET restaging was performed after 3 PE cycles, with the evidence of the persistent thickening and uptake within the tongue base. The patient underwent a new biopsy of the nasopharynx and base of the tongue with no tumour observed. The chemotherapy program was completed with mild acute toxicity (grade 2 alopecia ad grade 1 asthenia according to CTCAE v 4.0). A re-evaluation with functional and anatomic imaging (CT-PET scan) was carried out at the end of the CT program: complete remission (CR) was achieved. Thirty days after, the patients was planned to receive consolidation head and neck region radiation and PCI delivered with the TomoTherapy Hi-Art II system (TomoTherapy Inc,. Madison, WI) with the HA technique, as reported by Gondi et al. [10]. In order to evaluate basal neuro-cognitive functions, Mini Mental State Examination (MMSE) test was performed before radiation leading to a 30 out of 30 score. After proper immobilization (flat headboard and head-shoulders thermoplastic mask) and 2.5 mm slice thickness planning CT, target volumes and organs at risk contours were created within the Philips Pinnacle P3 v9.1 treatment planning system (Philips Medical System, Eindhoven, The Netherlands). The head and neck region volumes comprised the whole pharingo-laryngeal axis (from the roof of the naso-pharynx to the infra-glottic larynx) and the bilateral neck (level Ib to V and retro-pharyngeal nodes according to Robbins classification) with a 5 mm expansion from CTV to PTV to account for set up errors [9] (Figure 3a and 3c). The PCI volume comprehended the whole brain from the vertex to the occipital foramen (with the same 5 mm CTV to PTV expansion) (Figure 3a and 3c). For a correct delineation of the hippocampal regions, the patient underwent three-dimensional spoiled gradient axial magnetic resonance imaging (MRI) scans (3D-SPGR), standard axial and fluid attenuation recovery (FLAIR) scans and T2-weighted acquisitions, as suggested by Gondi et al. [10]. Semi-automatic rigid registration was performed between planning CT scans and MRI scans. The hippocampus was contoured on T1-weighted MRI axial sequences (T1-hypointense signal medial to the temporal horn) from the most caudal extent of the temporal horn to the lateral edges of the quadrageminals cisterns along the anterior-posterior axis (see Gondi et al. for details, [10]) (Figure 3b and 3d). A volumetrically isotropic 5 mm expansion was generated around the hippocampus to create the 'hippocampal avoidance volumes' (HAVs) for appropriate dose fall off between hippocampus and whole brain PTV (whole brain volume minus bilateral HAVs). Taking into account histology and complete remission status after induction chemotherapy, dose prescription was 60 Gy delivered in 30 fractions (2 Gy daily) for the head and neck region and 25.2 Gy in 14 fractions (1.8 Gy daily) for the whole brain PTV minus HAVs. The prescription dose was defined to the mean PTV and the 95% percentage PTV volume should be covered at least by 95% of the prescribed dose. In order to minimize late effects, conventional fractionation was employed for the 2 locations. Hence, the substantial difference in the number of fractions (30 vs 14) did not allow for the use simultaneous integrated boost (SIB) that would have lead to hypofractionation for the head and neck region. Therefore 2 different plans were generated. Isodose visualization was made importing both plans on the Oncentra Masterplan v 3.0 software (Nucletron, Veendhal, The Netherlands), since Tomotherapy does not allow for visualization of summed plans. Inverse planning algorithm constraints for head and neck regions organs at risks were as suggested by the Quantitative analysis of normal tissue effects in the clinic (QUANTEC) [11-14]. Dose constraints for the hippocampus (maximum dose 6 Gy and V_3 _≤ 20%) and HAVs (maximum dose 25.2 Gy and V_20 _≤ 20%) were adapted from Gondi et al. [10]. Metrics employed for tomotherapy planning were field width (FW) 2.5 cm, pitch 0.287, modulation factor (MF) planned 3.0 (actual 2.105) for the head and neck region and FW 1 cm, pitch 0.215, MF planned 3.2 (actual 2.7799) for whole brain radiation. Directional blocking was used only for lenses. The so obtained dose distribution is shown in Figures 4, 5. Dosimetric parameters are shown in Table 1. Radiation treatment was well tolerated with mild acute toxicity (grade 1 oral mucositis, skin reaction and xerostomia according to RTOG toxicity scale). No treatment interruptions occurred. Post-treatment re-evaluation showed complete remission at morphological and functional imaging with one year follow up. Grade 1 LENT-SOMA xerostomia could be detected as the only radiation-induced sequelae. Finally, MMSE results were unchanged compared to baseline.

**Table 1:** Dosimetric parameters overview

OARs	Dosimetric constraints	Dosimetric results	
*R hippocampus*	D_max _< 6 Gy V_3 Gy _< 20%	D_max _9,9 Gy	D_mean _6,5 Gy
			
			Median dose 6,3 Gy

*L hippocampus*	D_max _< 6 Gy V_3 Gy _< 20%	D_max _10,0 Gy	D_mean _6,5 Gy
			
			Median dose 6,2 Gy

*R HAV*	D_max _< 25 Gy V_20 Gy _< 20%	D_max _22,4 Gy	V_20 Gy _2,00%

*L HAV*	D_max _< 25 Gy V_20 Gy _< 20%	D_max _23,5 Gy	V_20 Gy _3,00%

*R lens*	D_max _< 6 Gy	D_max _4,8 Gy	

*L lens*	D_max _< 6 Gy	D_max _4,7 Gy	

*R ocular bulb*	D_max _< 54 Gy D_mean _< 35 Gy	D_max _17,8 Gy	D_mean _6,8 Gy

*L ocular bulb*	D_max _< 54 Gy D_mean _< 35 Gy	D_max _15,6 Gy	D_mean _6,3 Gy

*R optic nerve*	D_max _< 54 Gy	D_max _25,9 Gy	

*L optic nerve*	D_max _< 54 Gy	D_max _25,6 Gy	

*Optic chiasm*	D_max _< 54 Gy	D_max _28,3 Gy	

*Spinal cord*	D_max _< 45 Gy	D_max _28,6 Gy	

*Brainstem*	D_max _< 54 Gy	D_max _39,4 Gy	

*Oral cavity*	D_mean _< 45 Gy	D_mean _40,7 Gy	

*R cochlea*	D_mean _< 35 Gy	D_mean _38,1 Gy	

*L cochlea*	D_mean _< 35 Gy	D_mean _35,1 Gy	

*Pituitary gland*	D_max _< 40 Gy D_mean _< 35 Gy	D_max _29,9 Gy	D_mean _28,1 Gy

*Glottic larynx*	D_mean _< 50 Gy V_60 Gy _< 45%	D_mean _58,7 Gy	V_60 Gy _47,00%

*Mandible*	D_max _< 70 Gy D_mean _< 60 Gy	D_max _60,5 Gy	D_mean _45,6 Gy

*R parotid*	D_mean _< 26 Gy V_30 Gy _< 50%	D_mean _28,0 Gy	V_30 Gy _35,50%

*L parotid*	D_mean _< 26 Gy V_30 Gy _< 50%	D_mean _25,9 Gy	V_30 Gy _28,50%

*R brachial plexus*	D_max _< 55 Gy	D_max _50,6 Gy	

*L brachial plexus*	D_max _< 55 Gy	D_max _49,5 Gy	

*R lung*	V_45 Gy _< 33%	V_45 Gy _1,00%	

*L lung*	V_45 Gy _< 33%	V_45 Gy _1,50%	

*Thyroid*	V_30 Gy _< 50%	V_30 Gy _67,00%	

*R TMJ*	D_max _< 70 Gy D_mean _< 60 Gy	D_max _45,6 Gy	D_mean _27,0 Gy

*L TMJ*	D_max _< 70 Gy D_mean _< 60 Gy	D_max _44,0 Gy	D_mean _28,2 Gy

*OARs *organs at risk; *R *right; *L *left; *TMJ *temporo-mandibular joint

## Discussion

NSNECs of the head and neck region are widely uncommon and therefore clinical and therapeutic informations are scanty. In addition, the issue is beclouded by the slenderness of the published literature (mainly available throughout anecdotal reports) and by the heterogeneity of the histological sub-types and anatomical sites of presentation of the medical cases described. However some informative studies are available. To our knowledge, the largest case series of NSNECs published is the one by the MD Anderson Cancer Center: 23 patients were treated between 1984 and 2001 (median age 64 years; mainly smokers; predominant laryngeal primary tumours; locally advanced disease at diagnosis) [1]. The cohort underwent different treatment strategies including surgery, radiation and chemotherapy (in different combinations). With a median follow up of 40 months, 2-year and 5-year overall survival (OS) rates were 53% and 33%, respectively, while corresponding disease free survival (DFS) were 41% and 25%. Interestingly, since NSNEC is highly responsive to CT, the Authors reported that the inclusion of a DDP and VP-16 chemotherapeutic regimen in the multimodality treatment approach approximately doubled the 2 year OS and DFS. The most common pattern of failure is distant metastasis (DM) with a 2-year and 5-year rate of 54% and 71% respectively. The addition of CT in the therapeutic strategy reduced by one-half (79% vs 39%; *p *= 0.006) the 2-year rate of DM if compared to local therapy alone (either in univariate and multivariate analysis) [1]. Among DMs, intracranial spread often occurs with a 2-year and 5-year rate of 25% and 44% respectively. Moreover, isolated brain metastasis are quite frequent (21% and 41% of 2-year and 5-year rates). Local failure (LF) is infrequent (2-year and 5 year rate of 23%), specifically almost half of the frequency of comparably staged squamous cell carcinoma of the head and neck region [15]. Radiation therapy dose (range 44-72 Gy) did not correlate with LF (*p *= 0.23). CT did not prevent from LF (*p *= 0.91); however half of the patients with LF did not achieve complete remission (CR) after induction CT. Thereby, some general conclusions might be drawn. Surgical approaches should be limited to well-differentiated neuroendocrine carcinoma histological subtypes (typical carcinoids or carcinoid-like tumours), as it is for other body districts. Combined modality treatment consisting of chemotherapy and local radiotherapy should be strongly considered for moderately and poorly differentiated NSNECs. The preferable timing of the CT-RT combination is the sequential approach. Even if concurrent chemo-radiation has reached satisfactory evidence over sequential chemo-radiation in SCLC, induction CT and subsequent consolidation RT for complete or very good partial responders might be considered an efficient and less toxic approach for NSNECs, since concomitant CT-RT has not proven to improve early complete response rate, local control or survival [1]. However if macroscopic residual disease is present after induction CT, thereafter concurrent CT-RT or salvage surgery should be considered, since local control become the predominant clinical issue. The high rate of isolated brain metastasis is consistent with the fact that central nervous systems might harbour microscopic disease at diagnosis, thus calling for the need of eventual PCI. Generally, PCI has an established role in preventing the disabling symptoms due to intracranial metastasis and gives a survival benefit for patients affected with SCLC gaining intra-thoracic CR after combination therapy [16,17]. The aforementioned evidence might be translated in the clinical setting of NSNECs, considering the high risk of brain spread, suggesting the option of PCI for patients achieving CR after induction CT. Hence, the radiation strategy for this subset of patients might consist in large treatment volumes irradiated at first with a combination of PCI (dose range of 25-30 Gy delivered with conventional fractionation to reduce late effects) and consolidation RT to the head and neck region (primary site of tumour and corresponding draining lymphnodes) and a subsequent prosecution to head and neck only up to 60-70 Gy according to the appropriate clinical context. This approach supposedly avoids concerns regarding field junctions and isodose overlapping. Helical tomotherapy is particularly well-suited for this type of treatment since it is constituted by a continuously rotating, helical fan beam carved by a binary multileaf collimator mounted on a ring gantry that rotates around the treatment couch as it slowly progress within the gantry bore, through the beam delivery plane: therefore the length of the target volume does not represents a limiting factor since the equipment is able to proceed spirally around the patient for distances up to 160 cm [18,19]. PCI, as other typologies of cranial irradiation, might cause some grade of neurocognitive toxicity: late toxicity is described in long-term brain metastasis survivors submitted to whole brain radiotherapy in terms of cognitive deterioration and cerebellar dysfunction [20]. Moreover and early component of neurocognitive decline, involving verbal and short-term memory recall, has also been described with 1-4 months from WBRT for brain metastasis, regardless of response to treatment (diversely than executive and fine motor functions) [8]. Since the hippocampus has a crucial role in supporting memory function, its sparing possibly allows for a minimization of radiation-induced cognitive late effects, with possibly no detrimental effects on local control given the fact that the vast majority of brain metastasis arise beyond > 5 mm from the hippocampal region [21]. The hypothesis of a possible neurocognitive benefit of hippocampal avoidance in presently being tested by the RTOG within a Phase II prospective trial (namely RTOG 0933) which evaluates the effects on onset, frequency and severity of neurocognitive disorders in patients undergoing whole brain radiotherapy with concomitant hippocampus sparing for intracranial metastasis [8]. Given all the aforementioned background we chose to treat our patient (who achieved CR after induction CT) with consolidation radiation to the head and neck region and simultaneous PCI with the HA technique. The first 14 fraction were delivered both to the whole brain and head and neck region (a total of 25.2 Gy and 28 Gy respectively), while the remaining 16 fractions (2 Gy daily) were only delivered to the head and neck that received up to 60 Gy (2 different plans were generated). Planning and optimization were absolutely challenging, since dosimetric constraints to the hippocampus revealed hard to be respected due to the dosimetric contribute given by the head and neck region receiving 60 Gy and located only few centimetres below: thus bilateral hippocampus received a maximum dose of 7 Gy (instead of the planned 6 Gy), but the fact might be mitigated by the conventional fractionation employed. Even though it has been suggested that MMSE might have low sensitivity and specificity for testing neurocognitive function in patients affected with brain metastasis (conversely being well-suited for dementia evaluation) if compared to other examinations such as Hopkins Verbal Learning Test (HVLT), we chose this text in order to have a simple, agile and generally reliable metric to assess neurocognition [22,23]. At last the whole combined modality approach gave excellent short-term results in terms of tumor control and treatment-related toxicity.

## Consent

Written informed consent was obtained from the patient for publication of this case report and any accompanying images. A copy of the written consent is available for review by the Editor-in-Chief of this journal.

## Competing interests

The authors declare that they have no competing interests.

## Authors' contributions

PF, GN, FM, DC, PS, PC, MR LP, GG: provided medical assistance to the patients and defined the treatment approach; PC, PC, VCB: contributed in the treatment planning; PC: provided pathological specimens; PF, GN, DC, VCB, MP: contributed in the acquisition, analysis, interpretation of data; PF, GN: drafted the manuscript; ST, FO, UR: contributed in the critical revision; UR: gave final revision and approval. All authors read and approved the final manuscript.
